# Obituary

**Published:** 1847-06

**Authors:** 


*Obituary.—Prof. John P. Revere, and Geo. M'Clellan.*

The death of these distinguished individuals cannot but be felt primarily by the medical profession, and with reflex agency by the community at large. Although distinguished men in other professions may occupy a more prominent stand-point in the theatre of active life, and their disappearance from its stage be more speedily noted by the masses, yet if we would but look behind the closing curtain, there would be seen men who in a quiet, unostentatious life, silently, gradually, yet with more certainty have combatted moral and physical ills, though unheralded by the trump of fame.

We would not have the latter clause of these remarks applied rigidly to the individuals whose death we note, but make record of this truth in behalf of the cultivators of the medical profession at large, whose labor of duty and love seldom can appeal effectively but to the hearts of the restricted circle in which they move.

By repeated conversations, of thave we reiterated, that of all Lecturers on Practice of Medicine which it has been our privilege to hear, Prof. Revere took rank decidedly at the head. Having received such medical education in this country as the then limited opportunity afforded, by want of schools and able teachers, he was not content to *visit* Europe solely, but repaired to Edinburgh for *study*, at a time when its schools were deservedly in highest repute, entered the lists, and by diligent application bore off its honors, not for purpose of repose on these laurels, but with studious habits confirmed, ever after devoting himself to the culture of that profession to which he was ardently attached, and to the dissemination of its lore to the many willing listeners who gathered about his chair at whichever school he held a professorship.

There was nothing ornate in his style of composition or mode of teaching, but the hearer was held enchained by the earnest deep sense of responsibility which his manner and utterance conveyed, conjoined with that enthusiastic fervor which is ever the best outward manifestation of a sincere and ardent attachment to any pursuit.

Having thus labored for the love of truth and the cause of humanity, the sun of this scholar and christian is now set, but the light of his teachings have not set with him, but its rays are glowing in the hearts of his pupils and warm admirers wheresoever scattered abroad; and, so numerous are these, that few indeed are the large towns in our Union which are not represented by them, and yet less in number are those who will not join in saying,—not to have listened to his teachings would have been a loss irreparable. In short, Professor R. was a ripe scholar, whole-souled. and a good christian. Thus well fitted for a true life on earth, he has been borne hence to reap the reward laid up in store for him.

Prof. M'Clellan on entering the professional course singled out more particularly the department of Surgery for his pursuit, and led the way in after life to some of its most renowned operations. With indomitable energy he met all opposition that would stay his advancement, and that opposition by rivals was often acrimonious, not to say bitter—but in despite of all he pressed on to the goal. His characteristic trait was great decision, and activity, mental and physical, exhibited when engaged in private or professional duty, or as a teacher of Surgery. With a well stored mind, and a limited command of language, his conversation was helped on by pantomine, which, while lecturing to his class, forced the similitude of a "caged lion," ever moving, majestic, zealous and energetic. Popular with his class, he urged them forward to strive for the highest professional rank, and, to endeavor for all which is attainable, without truculent bending to the right or left to meet the views of any one who had caught the popular aura undeservedly. This inculcation perhaps had its origin in the severe school of his experience, and contrasts singularly with the teachings of a noted professor of Surgery, who when remarking on capital operations, with a gusto, adds;—"But gentlemen these operations you are to leave to the older members of the profession!" In short Prof. M'Clellan by precept and example, taught the great truth that success in Surgery, as in everything else, comes from well-grounded principles, and that manly self-reliance which prompts to individual action. With his large circle of admirers actively engaged in the profession, now remain his teaching and example—with these and their right application, they may well go forth, bidding the blind to see, the lame to walk, and the lepers be cleansed. W. T.

